# RhoA Promotes Synovial Proliferation and Bone Erosion in Rheumatoid Arthritis through Wnt/PCP Pathway

**DOI:** 10.1155/2023/5057009

**Published:** 2023-11-01

**Authors:** Ning Chen, Chao-Yue Diao, Xin Huang, Wei-Xing Tan, Ya-Bing Chen, Xin-Yu Qian, Jie Gao, Dong-Bao Zhao

**Affiliations:** ^1^Department of Rheumatology and Immunology, Changhai Hospital, Naval Medical University, Shanghai, China; ^2^Department of Rheumatology and Immunology, The First People's Hospital of Yancheng, The Fourth Affiliated Hospital of Nantong University, Yancheng, China; ^3^Department of Orthopedics, Huashan Hospital, Fudan University, Shanghai, China; ^4^Air Force Health Care Center for Special Services, Hangzhou, China

## Abstract

Ras homolog gene family member A (RhoA) plays a major role in the Wnt/planar cell polarity (PCP) pathway, which is significantly activated in patients with rheumatoid arthritis (RA). The function of RhoA in RA synovitis and bone erosion is still elusive. Here, we not only explored the impact of RhoA on the proliferation and invasion of RA fibroblast-like synoviocytes (FLSs) but also elucidated its effect on mouse osteoclast and a mouse model of collagen-induced arthritis (CIA). Results showed that RhoA was overexpressed in RA and CIA synovial tissues. Lentivirus-mediated silencing of RhoA increased apoptosis, attenuated invasion, and dramatically upregulated osteoprotegerin/receptor activator of nuclear factor-*κ*B ligand (OPG/RANKL) ratio in RA-FLSs. Additionally, the silencing of RhoA inhibited mouse osteoclast differentiation in vitro and alleviated synovial hyperplasia and bone erosion in the CIA mouse model. These effects in RA-FLSs and osteoclasts were all regulated by RhoA/Rho-associated protein kinase 2 (ROCK2) and might interact with Janus kinase/signal transducer and activator of transcription (JAK/STAT) pathways.

## 1. Introduction

Rheumatoid arthritis (RA) is a chronic inflammatory autoimmune disease that primarily affects small joints and is characterized by synovial inflammation and proliferation, cartilage erosion, and bone destruction [1]. Synovial hyperplasia is a central pathological change of RA and the main factor to the formation of an invasive pannus [2]. In RA, synovial hyperplasia is mainly caused by the proliferation of fibroblast-like synoviocytes (FLSs) and the infiltration of adaptive immune cells, including T cells, B cells, and macrophages [3, 4]. Of the potential cellular participants in RA, rheumatoid FLSs contribute to the production of proinflammatory cytokines and matrix metalloproteinases (MMPs), which degrade the extracellular matrix. In addition, FLSs develop unusual proliferative and aggressive phenotypes inexorably linked to cancer cells, but the precise etiology is still not known [5, 6].

Bone erosion is another important pathological feature of RA, and its severity is parallel to disease severity because it deteriorates the functional capacities of patients. Activated osteoclasts (OCs) at the interface between pannus and bone are the only cell type responsible for articular RA bone erosion [7, 8]. In spite of severe synovial inflammation and cartilage destruction, the progression of bone erosion is slow in RA patients with osteosclerosis [9]. Such role of OC is indicated by impaired osteoclastogenesis in the mouse models of arthritis, which were fully protected from bone destruction despite synovial inflammation [10]. OCs differentiate from the myeloid monocyte/macrophage lineage orchestrated by the receptor activator of nuclear factor-*κ*B (RANK) and RANK ligand (RANKL). RANKL is highly expressed in the synovial tissues of patients with RA, and its specific receptor RANK is on mononuclear OC precursors [11]. Osteoprotegerin (OPG) is a soluble decoy receptor of RANKL and can competitively inhibit the RANKL-RANK binding, and, thus, OC differentiation and activation are suppressed [12]. Conventional antirheumatic drugs for RA seem to have bone-sparing effects simply by effectively alleviating synovitis. Thus, the progression of bone erosion can still occur in patients with RA even with clinical remission because of residual synovitis and osteitis [13].

Wnt signaling cascades have essential roles in cell differentiation, proliferation, migration, and tissue homeostasis and are involved in RA pathogenesis [14, 15]. The canonical Wnt signaling in synovium is activated during RA development, resulting in synovial hyperplasia, inflammatory cell infiltration, and pannus formation [16]. Moreover, the noncanonical Wnt/PCP pathway not only participates in the activation of RA-FLS and the expression of RANKL [17] but also is implicated in the differentiation and activation of OCs [18]. In the Wnt/planar cell polarity (PCP) pathway, Wnt5a binds to its Frizzled receptor and receptor tyrosine kinase-like orphan receptor 2/related to tyrosine (Y) kinase (Ror2/RYK) coreceptors to recruit Dishevelled (Dvl), thereby triggering to the activation of Rho GTPases and downstream molecule Rho-associated protein kinase (ROCK) or c-Jun N-terminal kinase (JNK) [19]. Wnt5a is overexpressed in RA-FLS, and noncanonical Wnt5a signaling contributes to the aggressive phenotype of RA-FLS via the Wnt/Ca^2+^ and Wnt/PCP signaling pathways coupling with p38, ERK, and PI3K/AKT signaling [18]. In addition, Wnt/PCP signaling pathway is implicated in systemic and localized bone loss in patients with RA [20].

RhoA belongs to the Rho family GTPases [21]. It is a key component of the Wnt/PCP signaling pathway and significantly upregulated in RA serum exosomes [22]. RhoA acts as a molecular switch that regulates the activation of cytoskeletal proteins, and is pivotal for innate and adaptive immunity cell activation and migration [21]. ROCK, as a key downstream effector of RhoA, belongs to a family of the serine/threonine kinase and has two paralogs (ROCK1 and ROCK2) encoded by two different genes [23]. Previous studies on RhoA/ROCK were mainly concentrated on the area of cancer [24], and few studies in RA or inflammatory arthritis animal models were conducted. Here, the effect and mechanism of RhoA/ROCK on the biological phenotype of RA-FLS and mouse OC differentiation in vitro were investigated, and its role on the collagen-induced arthritis (CIA) model in vivo was demonstrated. Our study may provide a potential basis for novel therapeutic targets for ameliorating synovial inflammation and repairing bone erosion simultaneously in RA.

## 2. Materials and Methods

### 2.1. Patients Enrollment and Synovial Tissue Preparation

Synovial tissues were collected from six patients with RA (two cases from arthroscopic operation and four cases from knee replacement surgery) and six patients with knee injury (osteoarthritis and inflammatory arthritis were excluded by arthroscopy). The inclusion criteria for RA were based on the 2010 American College of Rheumatology/European Alliance of Associations for Rheumatology (ACR/EULAR) classification criteria [25]. This study was approved by the Institutional Ethics Committee of the Changhai Hospital, Shanghai, China.

### 2.2. Immunohistochemical of Synovial Tissue and Histopathology of Mice

The synovial tissues of joints were immersed in 4% paraformaldehyde for 48 hr and then analyzed by immunohistochemistry (IHC). The IHC scores were in calculating the percentage of RhoA positive cells (0 = 0%−9%, 1 = 10%−24%, 2 = 25%−49%, 3 = 50%−74%, 4 = 75%−100%) and staining strength (0 = unstained, 1 = light yellow, 2 = brown-yellow, 3 = brown) according to the standards of previous studies [26, 27].

The knee joints of mice were fixed in 4% paraformaldehyde for 72 hr, decalcified in 14% EDTA-glycerol for 4 weeks at 37°C, and embedded in paraffin. Immunohistochemical staining was performed according to previous methods. Hematoxylin and eosin (H&E) and tartrate-resistant acid phosphatase (TRAP) staining were performed for the examination of synovitis and OC infiltration. The severity of arthritis in CIA mice was assessed according to the H&E scoring standard [28]. The number of TRAP positive cells per unit area of the joint synovium was determined.

### 2.3. RA-FLSs Isolation and Culture

The synovial tissues were cut into 1 mm^2^ pieces and digested in type II collagenase solution (1 mg/ml) at 37°C constant temperature shaker for 4 hr. The cells were cultured in Dulbecco's Modified Eagle Medium (DMEM) supplemented with 10% fetal bovine serum (FBS) (Gibco, USA) at 37°C and 5% CO_2_ humidified incubator. They were harvested for experiments at three to six passages [27]. For the identification of isolated FLSs, the expression levels of CD68 (negative) and vimentin (positive) were detected by immunofluorescence.

### 2.4. BMMC Isolation and OC Induction

Mouse marrow cavity was rinsed with a-MEM, and then the marrow flushing solution was filtered by a cell filter (200 *µ*m mesh). RBC lysis solution was added to remove RBCs. The cells were resuspended using a-MEM (containing 10% FBS) and cultured at 37°C in a 5% CO_2_ humidified incubator. The next day, unadherent cells were collected using culture medium containing 30 ng/ml M-CSF (PeproTech, USA) and were harvested when the cell density was 80%. Bone marrow mononuclear cells (BMMCs) were inoculated into 12-well plates (1 × 10^5^/well), and 30 ng/ml MSCF and 100 ng/ml RANKL (PeproTech, USA) were added to the culture medium to induce OC formation. The medium was changed regularly. RNA and protein were extracted for the detection of RhoA expression before induction and on the 3rd and 5th day of induction.

### 2.5. Immunofluorescence

The distribution of RhoA in the FLSs of two groups was assessed by immunofluorescence, which was performed as described previously [29]. After staining, the cells were then visualized and imaged with a fluorescence microscope (Olympus, Tokyo, Japan) [27].

### 2.6. Cell Infection

Lentiviruses encoding RhoA (siRNA in human and mouse cosource region) was constructed and produced by Jikai Technology (Shanghai, China). The RA-FLSs were cultured in six-well plates for 24 hr, and the cell density was 20%–30% on the next day. According to the multiplicity of infection (MOI) obtained in the preexperiment, the venom was prepared and added to the six-well plates gently. After 8 hr, the liquid was changed. The best MOI for Sh-Ctr and Sh-RhoA was 10 and 20, respectively. Then, 72 hr after lentivirus infection, 4 *μ*g/ml puromycin (Sigma-Aldrich, USA) was added to remove wild cells, and a follow-up experiment was conducted 1 week later. Western blot was performed to determine the efficiency of infection.

BMMCs were inoculated into six-well plates, and the density of the cells was about 40% on the next day. The MOI of control and Sh-RhoA lentivirus was both 10. Seventy-two hours later, the transfection effect was observed with fluorescence microscopy, and qPCR was used in determining virus transfection efficiency.

### 2.7. Cell Migration

Transwell cell migration test and wound healing assay were used in determining the migration ability of RA-FLSs after lentivirus infection. Transwell invasion chambers (Corning, USA) were placed in 24-well plates, and the numbers of RA-FLSs in five randomly selected fields under the microscope in each well were calculated according to a previously described method [30]. The cell migration rate was determined as the average numbers of invading cells compared to the Sh-Ctr group.

The transfected cells were inoculated into six-well plates. The wound healing process was analyzed by phase contrast microscopy (Leitz, Germany) at 0 and 24 hr, according to a previous report [30]. The area of cell migration at different time points was calculated by Image J software. Mobility = (0 hr scratch area – 24 hr scratch area)/0 hr scratch area [27].

### 2.8. Measurement of Apoptosis

Apoptosis was assessed with a one-step tunel apoptosis detection kit (Beyotime, China). Briefly, serum-free medium was added to infected RA-FLSs to induce apoptosis, and then the cells were fixed with formaldehyde 48 hr later. This procedure was followed by treatment with TdT enzyme and fluorescent-labeled solution for 60 min at 37°C in the dark. The nucleus was stained with Hoechst solution, and the apoptosis rates of the two groups were compared under a fluorescence microscope.

### 2.9. Cell Viability Assay

The impact of Sh-RhoA on cell viability was assessed using Cell Counting Kit-8 (CCK-8) (Dojindo, Japan). The infected RA-FLSs were incubated in 96-well plates (3 × 10^3^ cells/well) for 24 or 48 hr, and then 10 *μ*l of CCK-8 solution and 100 *μ*l of DMEM/well were added. After incubation at 37°C for 4 hr, absorbance was measured at 450 nm wavelength with a spectrophotometer [27].

### 2.10. TRAP Staining of OC

The BMMCs were inoculated into 12-well plates with about 1 × 10^5^ cells/well, and 30 ng/ml MSCF and 100 ng/ml RANKL were added to the culture medium for induction. Five days after induction, TRAP staining was performed according to the standard protocol (Sigma-Aldrich), and changes were photographed under a microscope.

### 2.11. Dil Cell Membrane Fusion Experiment

BMMCs in blank, Sh-Ctr, and Sh-RhoA group were inoculated into new 12-well plates for induction. On the 3rd day of induction, the cell supernatant was discarded, and the cells were washed three times with prewarmed a-MEM. Dil working solution (1 *μ*l of Dil and 10 ml of a-MEM) was then added. The cell dish was shaken gently and placed in the incubator at 37°C for 30 min. The Dil solution was removed, and the cells were washed three times and resuspended with a new culture medium containing M-CSF and RANKL. The cells were counted and then inoculated into 12-well plates again for further culture. After 24 hr, the BMMCs were fixed for 10 min with formaldehyde, and the nucleus was stained with Hoechst solution. Changes were observed and photographed under a fluorescence microscope.

### 2.12. Animal Studies

We used three normal mice (DBA/1) in exploring the dose of the virus. After injecting lentivirus into mouse knee joint (once a week) for 3 weeks, we performed IHC to detect the expression of the enhanced green fluorescent protein (EGFP) gene carried by the lentivirus. The DBA/1 male mice were 8 weeks old (they had been kept in the SPF animal center of our hospital for 1 week to adapt to the environment) and purchased from SLRC Laboratory Animals (Shanghai, China). All animal procedures and handling were approved by the Animal Care and Use Committee of Changhai Hospital (Shanghai). A total of 18 mice were randomly divided into three groups: Sh-Ctr, Sh-RhoA, and normal control groups, and CIA was established as previously described. The lentivirus was administered into the knee joints of mice 28 days after the first immunization. Clinical arthritis can be usually observed at this time. After the second immunization, the severity of arthritis was scored every other day [31], and scoring was independently conducted by two people. The final average was obtained. Finally, on the 54th day, the knee joints were stained by IHC, H&E, and TRAP.

### 2.13. Collagen-Induced Arthritis Model

Use a handheld grinder (Northern Tool Equipment, Ningbo, China) to emulsify 4 mg/ml complete Freund's adjuvant (Chondrex, Redmond, WA, USA) with 2 mg/ml bovine type II collagen solution (Chondrex) at ratio of 1 : 1 until the obtained liquid had a milky white appearance and was insoluble in water. The whole emulsification process required to be performed on ice to prevent heating. Each mouse was injected with 100 *μ*l of the emulsion subcutaneously in the tail. The booster injection was performed on the 21st day with the same method, while an emulsion of incomplete Freund's adjuvant (Chondrex) and bovine type II collage was recommended. For more specific directions, please refer to this published protocol [31].

### 2.14. Micro-CT

The micrographs of the subchondral bone of the mice knee were obtained using micro-CT system (Skyscan 1172) at 16 *μ*m isotropic voxel size. After scanning, CT-Vol software (version 1.14) was used for three-dimensional analysis.

### 2.15. ELISA Assay

The venous blood of mice was collected, kept at room temperature for 30 min, and centrifuged at 1,000 *g* for 10 min. The supernatant was then collected. The levels of IL-17 and IL-21 were detected with an ELISA kit (R&D System, USA).

### 2.16. qPCR Assays

Total RNA was extracted from infected RA-FLSs and BMMCs by using TRIzol Reagent (Invitrogen) according to the manufacturer's instructions. cDNA synthesis and RT-PCR were performed according to protocols of the PrimeScript RT Reagent Kit and SYBR Green (Takara). The expression of GAPDH in the cDNA samples was used as the control. The primers used are shown in Tables S1 and S2. The 2^−*ΔΔ*Ct^ method was used in quantifying the relative mRNA expression levels.

### 2.17. Western Blot

Synovial tissue and cells were lysed using proper lysis buffer, and the proteins were collected after centrifugation. Quantitative protein samples were separated through sodium dodecyl sulfate-polyacrylamide gel electrophoresis and transferred to polyvinylidene fluoride membranes. The membranes were incubated overnight with appropriate antibodies. GAPDH antibodies (Sigma-Aldrich) were incubated as controls. After incubation with horseradish peroxidase (HRP)-conjugated secondary antibodies (CST), immunoreactive bands were visualized using chemiluminescence reagents (BIORAD). The catalog numbers of the regarding antibodies are shown in Table S3.

### 2.18. Immunoprecipitation

Third-generation RA-FLSs were harvested and lysed using NP40 lysate buffer with protease and phosphatase inhibitors (Beyotime, China). The supernatants were collected after centrifugation. Corresponding antibodies or IgG (negative control) and magnetic beads were added to the quantitative protein samples, which were then incubated overnight at 4°C on a rotary shaker. Immunological complexes were extracted and boiled, and western blotting was performed.

### 2.19. Statistical Analysis

All the data are from at least three independent experiments and expressed as the mean standard error. By using SPSS 26.0, differences between two were compared with Student's *t*-test, and three groups were compared with one-way analysis of variance (ANOVA), followed by a Tukey's multiple comparison posttest. All significant differences were considered at *P*-values < 0.05.

## 3. Results

### 3.1. RhoA Expression Increased in the Synovial of Patients with RA and CIA Mice

Our previous studies have described that RhoA is significantly upregulated in RA-derived serum exosomes [22]. To assess the expression of RhoA in patients with RA and CIA mice, we collected human synovial tissues from RA (*n* = 6) and trauma (*n* = 6) patients and joint samples from CIA (*n* = 6) and normal mice (*n* = 6).

**Figure 1:**
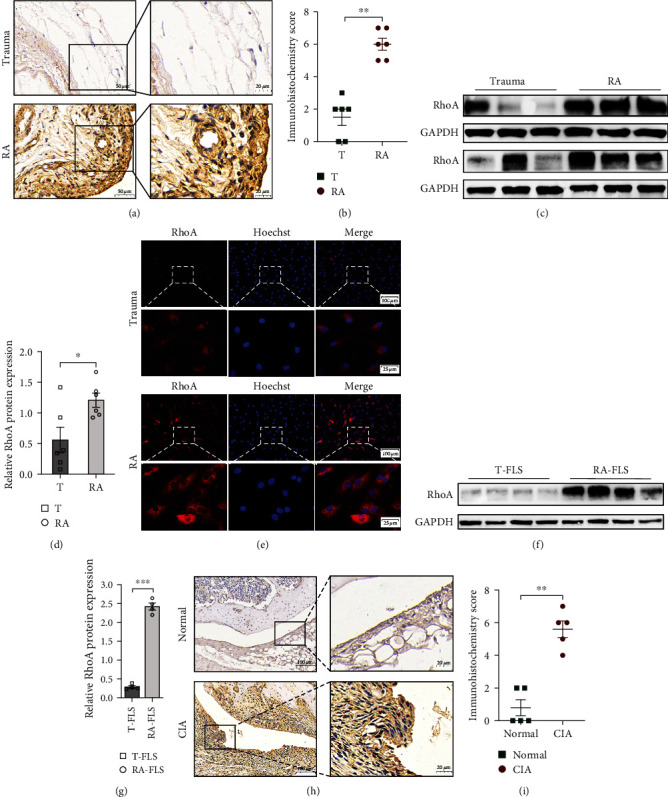
Validation of RhoA expression in the synovium and FLSs from RA and CIA mice. (a and b) IHC analysis tested the expression of RhoA in synovial extracts from trauma (*n* = 6) and RA (*n* = 6) patients, *p*=0.002. (c and d) Western blot results for RhoA and GAPDH of synovial tissues from trauma (*n* = 6) and RA (*n* = 6) group, *p*=0.035. (e) The immunofluorescence analysis of RhoA expression in the FLSs of RA and trauma patients (*n* = 4). (f and g) Western blot analysis which was used in detecting the expression of RhoA in trauma-FLSs and RA-FLSs (*n* = 4), *p* < 0.001. (h and i) RhoA IHC analysis of knee synovium from wide or CIA mice (*n* = 5), *p*=0.003.  ^*∗*^*p* < 0.05,  ^*∗∗*^*p* < 0.01,  ^*∗∗∗*^*p* < 0.001.

The IHC results (Figures 1(a) and 1(b)) confirmed that expression levels of RhoA were higher in synovial tissues from RA than in those from trauma patients, which were present as brown granules in the cytoplasm and nucleus. Then, we further examined the expression of RhoA in RA-associated synovial tissues through western blotting (Figures 1(c) and 1(d)), and similar results showed that it was 2.160-fold of the control. We performed immunofluorescence (Figure 1(e)) and western blotting (Figures 1(f) and 1(g)) to detect the level of RhoA in RA-FLSs and trauma-FLSs, and the results indicated that RhoA significantly increased in RA-FLSs compared with the control. A similar increase in RhoA expression was observed in the knee joints from CIA mice compared with wild-type mice by IHC analysis (Figures 1(h) and 1(i)).

### 3.2. Sh-RhoA Inhibited the Proliferation, Invasion, and Inflammation of RA-FLS and Upregulated the OPG/RANKL Ratio

**Figure 2:**
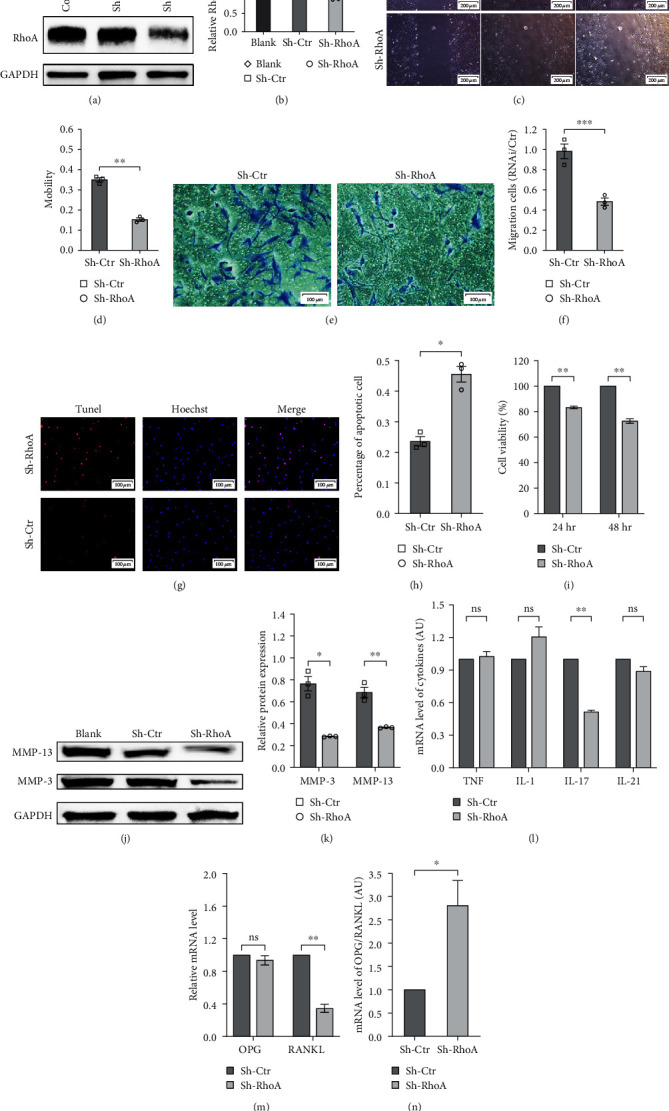
Impact of Sh-RhoA on migration, apoptosis, viability, and inflammatory response of RA-FLSs. (a) The effects of Sh-RhoA infection on protein expression. (b) Differences in the relative ratios of RhoA to GAPDH between three groups (*n* = 3); *p*=0.001, Sh-RhoA versus blank; *p*=0.006, Sh-RhoA versus Sh-Ctr. (c and d) The results of wound healing assay showed that the cell migration ability in the Sh-RhoA group was lower than that in the control group, *p*=0.003. (e) The results of Transwell cell migration test in RA-FLSs. (f) The number of migration cells in Sh-RhoA group was lower than that in the Sh-Ctr group, *p* < 0.001. (g) Representative images showing the Tunel + cells (red) of Sh-RhoA group and control. The nucleus was stained with Hoechst solution (blue). (h) The statistic percentage of Tunel + cells in indicated conditions, *p*=0.034. (i) The results of CCK-8 assay revealed that Sh-RhoA reduced the viability of RA-FLSs, *p*=0.004, 0.004. (j and k) Western blot analyses revealed that in the Sh-RhoA group, the expression of MMP-3 and MMP-13 was lower than that in the control group, *p*=0.017, 0.003. (l) The relative mRNA levels of TNF-*α*, IL-1*β*, IL-17, and IL-21. Sh-RhoA only restrained the secretion of IL-17 (*p*=0.616, 0.088, 0.001, 0.120). (m) The relative mRNA levels of OPG and RANKL. Sh-RhoA inhibited the level of RANKL in RA-FLSs (*p*=0.374, 0.006). (n) The ratio of OPG/RANKL increased significantly in the Sh-RhoA group (*n* = 3), *p*=0.028.  ^*∗*^*p* < 0.05,  ^*∗∗*^*p* < 0.01,  ^*∗∗∗*^*p* < 0.001.

Sh-RhoA lentivirus was transfected into RA-FLSs at an MOI of 20. After 48 hr of transfection, puromycin was added to the medium, and infected cells were collected. After 5 days, transfection efficiency was measured by western blotting (Figures 2(a) and 2(b)), and RhoA expression (a 0.432-fold decrease) was obviously inhibited in the RA-FLSs of interfering group.

Research has shown that RA-FLS possesses the ability of tumor-like invasion and proliferation, while apoptosis decreases significantly. Data from scratch wound healing (Figures 2(c) and 2(d)) and Transwell assay (Figures 2(e) and 2(f)) indicated that Sh-RhoA significantly inhibited the migration and invasion of RA-FLSs. By terminal deoxynucleotidyl transferase mediated dUTP nick-end labeling (TUNEL) assay (Figures 2(g) and 2(h)), we found that TUNEL-positive cells had a 1.929-fold increase in the Sh-RhoA group, revealing that RhoA had an important role in preventing apoptosis. CCK-8 assay (Figure 2(i)) was used in investigating the effects of RhoA on RA-FLS proliferation, and Sh-RhoA significantly restrained the growth of RA-FLSs. At 24 and 48 hr, the absorbance values of the Sh-RhoA group were 0.833 and 0.726 times of the control group, respectively. MMPs help RA-FLSs to invade articular cartilage, thus resulting in articular cartilage impairment [32]. Western blot (Figures 2(j) and 2(k)) revealed that Sh-RhoA dramatically decreased the expression of MMP-3 (0.370-fold) and MMP-13 (0.533-fold).

IL-17 is significantly upregulated in synovial fluids and synovial tissues [33, 34]. Therefore, we explored the inflammatory cytokines of RA-FLSs by qPCR (Figure 2(l)) and found that the expression of IL-17 mRNA was a 0.514-fold decrease after RhoA knockdown.

Previous studies on patients with RA and animal models have observed that decrease in the OPG/RANKL ratio can promote OC formation [17]. Our results (Figures 2(m) and 2(n)) confirmed that Sh-RhoA can lower the level of RANKL mRNA and increase the OPG/RANKL ratio.

### 3.3. Sh-RhoA Inhibited Differentiation of BMMCs into OCs

**Figure 3:**
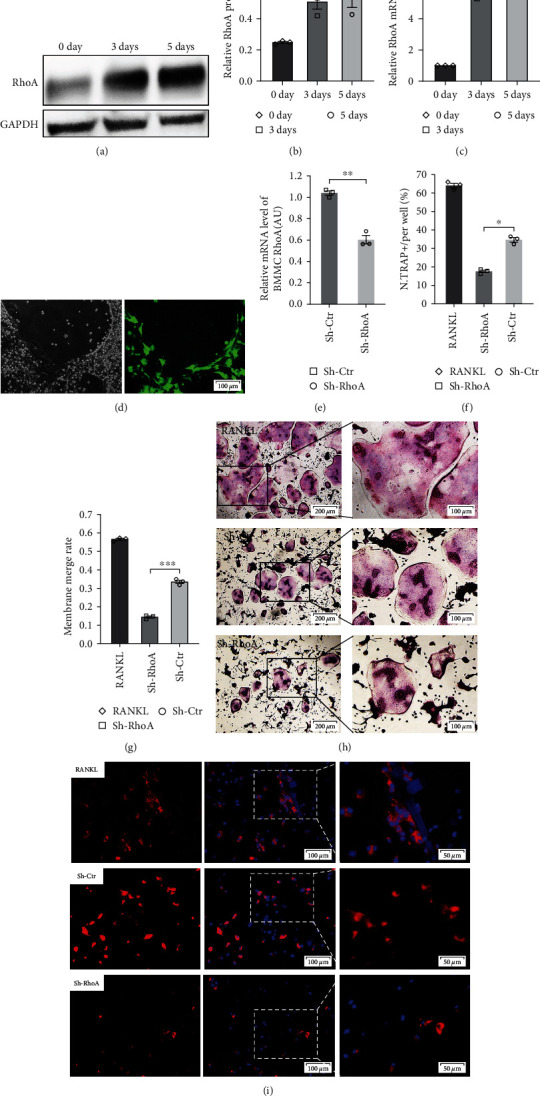
Expression of RhoA in OC differentiation and the effect of Sh-RhoA on OC formation. (a–c) Differences in the relative protein and mRNA levels of RhoA between three groups (*n* = 3); the relative protein (*p*=0.009, 3 days vs. 0 day) (a and b) and mRNA (*p*=0.002, 3 days vs. 0 day) (c) levels of RhoA enhanced obviously on the third day. (d) The typical fluorescence images of transfected BMMCs differentiated into OCs. (e) The level of RhoA mRNA was lower in BMMCs transfected with Sh-RhoA lentivirus, *p*=0.007. (f) Differences in the percentage of mature OCs between three groups; the percentage of mature OCs in the Sh-RhoA group was lower than that in the Sh-Ctr group, *p*=0.012. (g) Differences in the BMMC membrane fusion rate between three groups; the BMMC membrane fusion rate was significantly reduced in the Sh-RhoA group compared with the Sh-Ctr group, *p* < 0.001. (h) The results of TRAP assay. (i) Dil fluorescence staining of cell membrane during BMMC fusion (*n* = 3).  ^*∗*^*p* < 0.05,  ^*∗∗*^*p* < 0.01,  ^*∗∗∗*^*p* < 0.001.

BMMCs were extracted from the femoral bone marrow of C57BL/6 mice and stimulated with RANKL + M-CSF. RhoA protein and mRNA expression levels in the 0, 3th, and 5th day of induction were detected by western blotting (Figures 3(a) and 3(b)) and qPCR (Figure 3(c)), respectively. Our data revealed that RhoA expression was dramatically elevated (2.051-fold) on the 3rd day, which was maintained until the 5th day.

Mouse BMMCs were transfected with Sh-RhoA lentivirus at an MOI of 10, and transfection efficiency was measured using qPCR (Figure 3(e)) after 72 hr. Compared with the control group, the relative quantity of Rho mRNA expression was 0.581 ± 0.062 (*p*=0.007). BMMCs successfully transfected were stimulated with the same method.  Figure 3(d) shows the typical fluorescence images of transfected BMMCs that differentiated into OCs.

The TRAP assay (Figures 3(f) and 3(h)) was carried out to evaluate OC formation. The percentage of mature OCs in the Sh-RhoA group was lower than that in the Sh-Ctr group (*p*=0.012), suggesting that RhoA played a crucial role in OC differentiation. Given a significant increase in RhoA during the middle term of differentiation, we assessed BMMC membrane fusion by Dil staining on the 3rd day after the stimulation. The results (Figures 3(g) and 3(i)) revealed that BMMC membrane fusion rate was significantly reduced (0.144 ± 0.010 vs. 0.335 ± 0.016) in the Sh-RhoA group compared with the Sh-Ctr group.

### 3.4. Sh-RhoA Attenuated Synovial Inflammation, Bone Destruction, and Bone Loss in CIA Mice

**Figure 4:**
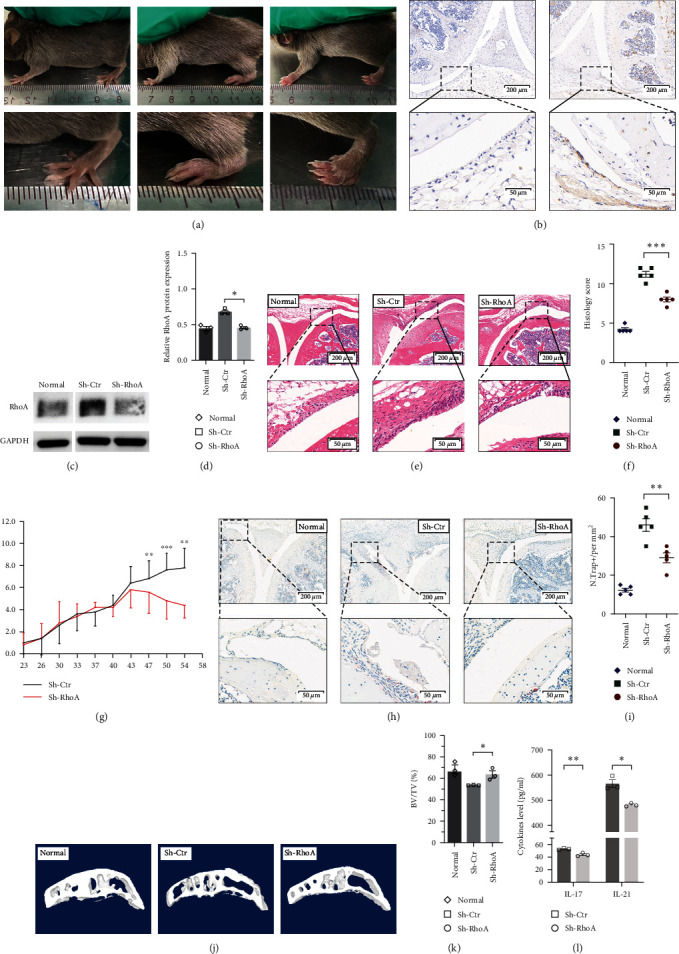
Effect of Sh-RhoA on CIA mice. (a) Joint changes in CIA mice. (b) The IHC analysis tested the EGFP expression to determine the transfected effect. (c and d) The western blot result showed that the expression of RhoA in the joint synovium was significantly downregulated in the Sh-RhoA group compared with the Sh-Ctr group (*n* = 3), *p*=0.017. (e) The typical HE changes in knee joint in the three groups (*n* = 5). (f) The H&E scores were significantly lower in the Sh-RhoA group than in the control group (*n* = 5), *p* < 0.001. (g) Compared with the Sh-Ctr group, the arthritis scores in Sh-RhoA group were significantly ameliorated after the third intra-articular injection (*n* = 5), *p*=0.004, *p* < 0.001, *p*=0.001. (h and i) TRAP staining on joints synovium showed that the Sh-RhoA group had fewer TRAP-positive cells (*n* = 5), *p*=0.004. (j and k) Micro-CT showed the influence of Sh-RhoA on the microstructure of subchondral bone in CIA mice. The results indicated that bone volume/tissue volume (BV/TV) was elevated in the Sh-RhoA group compared with the control group (*n* = 3), *p*=0.026. (l) The levels of IL-17 and IL-21 in mouse serum tested by an ELISA assay kit (*n* = 3), *p*=0.008, 0.040.  ^*∗*^*p* < 0.05,  ^*∗∗*^*p* < 0.01,  ^*∗∗∗*^*p* < 0.001.

To demonstrate the treatment effects of RhoA on RA in vivo, we established a CIA model (Figure 4(a)), in which lentivirus (1 × 10^9^ IU/ml) was injected into the stifle joints of mice. For transfection efficiency testing, EGFP expression levels were detected by IHC (Figure 4(b)). The western blot results (Figures 4(c) and 4(d)) confirmed downregulated RhoA expression in the synovial tissues of the stifle joint.

By H&E staining of the stifle joint from different groups, we showed that Sh-RhoA reduced inflammatory cell infiltration, synovial hyperplasia, pannus formation, and bone destruction (Figures 4(e) and 4(f)). The arthritis scores in the Sh-RhoA group were significantly ameliorated after the third intra-articular injection (day 47, *p*=0.004) (Figure 4(g)) compared with those in the Sh-Ctr group.

To further observe the role of RhoA on OC precursor and OC formation in synovial pannus and bone destruction sites in vivo, we performed TRAP staining on the stifle joints of mice. As shown in Figures 4(h) and 4(i), synovial tissues in the Sh-RhoA group had fewer TRAP-positive cells than those in the Sh-Ctr group, and the Sh-RhoA group displayed reduced levels of bone erosion and destruction. Micro-CT and 3D reconstruction showed the effects of RhoA on subchondral bone microstructure in CIA mice. The results showed that compared with Sh-Ctr, Sh-RhoA greatly reduced bone destruction, as quantitatively evidenced by the regularly and densely arranged bone trabeculae and a statistically significant increase in BV/TV (*p*=0.026).

To determine the effects of RhoA on the secretion of inflammatory cytokines in vivo, we investigated the expression of IL-17 and IL-21 in serum by ELISA. As shown in Figure 4(l), the expression of IL-17 and IL-21 in the Sh-RhoA group was lower than that in the Sh-Ctr group.

### 3.5. RhoA Regulates the Behavior of OCs and FLSs through ROCK2

**Figure 5:**
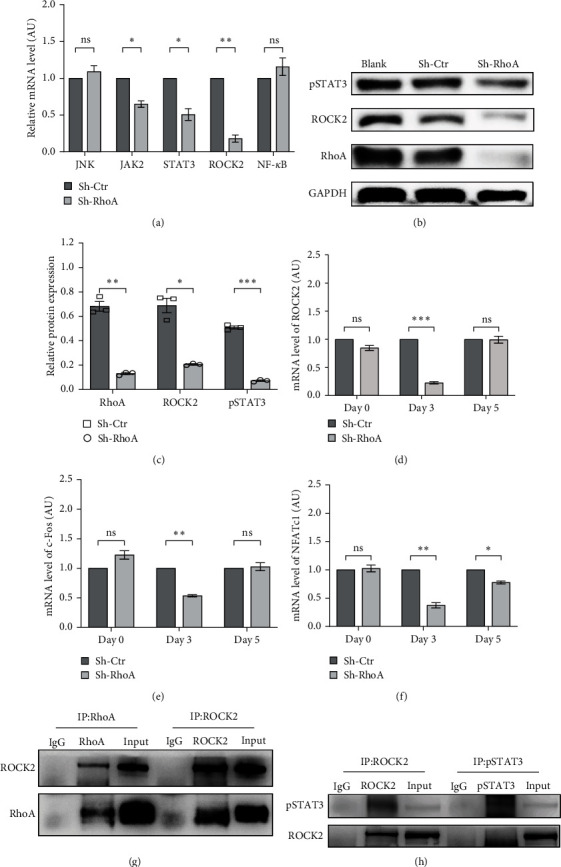
Mechanism research of RhoA involved in synovitis and bone erosion. (a) Relative mRNA expression levels of JNK, JAK2, STAT3, ROCK2, and NF-*κ*B in transfected RA-FLSs, *p* = 0.380, 0.016, 0.025, 0.004, 0.321. (b and c) The protein levels of RhoA (*p*=0.004), ROCK2 (*p*=0.013), and pSTAT3 (*p* < 0.001) in the RA-FLSs of indicated groups determined by western blot. (d–f) The mRNA levels of ROCK2 (d, *p* < 0.001), c-Fos (e, *p*=0.001), and NFATc1 (f, *p*=0.006, 0.014) in the process of transfected BMMCs differentiating into OC. (g) IP analysis showed that RhoA interacted with ROCK2 in RA-FLSs. (h) IP analysis showed that ROCK2 interacted with pSTAT3.  ^*∗*^*p* < 0.05,  ^*∗∗*^*p* < 0.01,  ^*∗∗∗*^*p* < 0.001.

The Wnt/PCP pathway plays a vital role in regulating synovial proliferation and bone remodeling [35, 36]. Through qPCR (Figure 5(a)), our studies found that Sh-RhoA remarkably suppressed the expression of ROCK2, Janus kinase 2 (JAK2), and signal transducer and activator of transcription 3 (STAT3) in RA-FLSs. Western blotting (Figure 5(a)) results confirmed that RhoA silencing can attenuate the expression of ROCK2 and p-STAT3.

RANKL mediates OC fusion and differentiation by inducing the expression of c-Fos and nuclear factor-activated T-cell 1 (NFATc1) [37]. Our qPCR (Figure 5(d)–5(f)) results revealed that Sh-RhoA significantly decreased the expression of ROCK2 (0.226-fold), c-Fos (0.534-fold), and NFATc1 (0.372-fold) mRNA in mouse OC precursor cells (BMMCs) 3 days after stimulation, whereas Sh-RhoA only decreased NFATc1 mRNA expression on the 5th day.

Co-immunoprecipitation (CO-IP) was used in studying the interactions between RhoA with ROCK2, ROCK2, and pSTAT3. Our results (Figures 5(g) and 5(h)) suggested that RhoA in RA-FLSs works through direct impacts on ROCK2, and RhoA/ROCK2 signal transduction can couple with the JAK/STAT pathway to regulate the behavior of RA-FLSs.

## 4. Discussion

RA is a chronic inflammatory autoimmune disease. Synovitis and bone erosion are the two main pathological features, and synovitis is the important cause of bone erosion, which is closely related to the RA severity and joint function status in RA [38]. Our previous study suggested that Dvl3 expression increased in the synovium and FLS of RA and significantly upregulated the expression of *β*-catenin and RhoA [22]. Meanwhile, the activation of different domains of Dvl3 in OCs can activate the Wnt/*β*-catenin and RhoA/ROCK pathways [39].

Our study showed that the expression of RhoA was significantly upregulated in the joint synovium of RA and CIA and in RA-FLSs, suggesting that the Wnt/PCP pathway is activated in RA and animal models of inflammatory arthritis. We observed that the expression of RhoA gradually increased during the differentiation of mouse BMMCs into OCs, especially on the 3rd day of induction process. This result suggested that RhoA plays a certain role in the differentiation of OC. We hypothesized that RhoA not only plays an important role in the pathogenesis of RA synovitis but also participates in the activation of OC, causing bone erosion and osteoporosis in RA.

To determine the effect of RhoA on the phenotype of RA-FLS, we transfected RA-FLSs with RhoA interference and control lentivirus. The results showed that Sh-RhoA significantly inhibited the migration and proliferation of RA-FLSs and inhibited the expression of MMP-3–13. MMPs are important causes of cartilage erosion [40]. Reduced apoptosis is an important feature of RA-FLSs [41]. Apoptosis is a controlled form of cell death [42]. The RhoA/ROCK signaling pathway plays an important role in apoptosis, but the proapoptotic or antiapoptotic effects mainly depend on cell type and surrounding microenvironment [43]. Our study first showed that the downregulation of RhoA expression in RA-FLSs plays a proapoptotic role.

The RhoA/ROCK pathway can regulate the differentiation of Th17 cells, and Th17 mainly secretes inflammatory factors IL-17 and IL-21 [33, 44]. IL-17 is highly expressed in the synovium of RA and plays an important role in local inflammation, and can promote the secretion of RANKL by RA-FLSs and induce the formation of OCs [34, 45]. The results of this study showed that the downregulation of RhoA only inhibited the expression of IL-17 in vitro, whereas sh-RhoA inhibited the secretion of IL-17 and IL-21 in the serum of CIA mice in vivo. This phenomenon suggests that RhoA directly participates in the metabolic process of IL-17, but other pathways may be involved in the secretion of IL-21. The specific mechanism still needs to be further studied.

OC is the only cell in the body that can absorb bone [46]. RANKL is essential for OC differentiation in humans and mice [47]. OPG is a soluble bait receptor for RANKL [48]. Decrease in serum OPG/RANKL ratio in newly diagnosed RA is an independent predictor of rapid joint destruction and sustained progression [49]. Our study showed that Sh-RhoA can inhibit RANKL expression, increase OPG/RANKL ratio in RA-FLSs, and significantly inhibit BMMC fusion, and the proportion of mature OC significantly decreased.

The study in vivo further verified the therapeutic effect of Sh-RhoA. Injection of Sh-RhoA lentivirus into the knee significantly reduced the joint score, synovial inflammation, infiltration of OC precursor cells, and secretion of inflammatory factors in mice. The subchondral bone volume fraction of the experimental group mice increased correspondingly.

After clarifying the effects of RhoA on RA-FLS, OC, and CIA models in vitro and in vivo, we further investigated the related mechanisms. The results of qPCR, western blot, and CO-IP showed that RhoA transduced signals through ROCK2 in RA-FLS and ROCK2 interacted with phosphorylated STAT3, which played a role in RA synovitis. In addition, Sh-RhoA can inhibit the expression of ROCK2, c-Fos, and NFATc1 mRNA in mouse BMMC. RANKL regulates OC differentiation mainly through c-Fos and NFATc1 [50]. c-Fos is the molecular basis of OC differentiation, and NFATc1 is the target gene of c-Fos [51]. Calcineurin inhibitors can effectively inhibit OC differentiation by inhibiting NFATc1 [52]. The results suggested that RhoA/ROCK2 had a direct effect on RANKL-induced OC differentiation.

Previous studies have shown that targeting ROCK2 can regulate the balance between Th17 and Treg to restore immune homeostasis [53]. Our study is the first to propose that RhoA/ROCK2 affects the biological behavior of RA-FLS and the differentiation of OC in mice. The JAK/STAT pathway represents a central pathway that mediates cellular responses to various cytokines and growth factors [54]. JAK inhibitor (JAKi) can significantly downregulate pSTAT3, and the correlation between STAT3 and Wnt pathways has been reported, which plays a synergistic role in cell survival and proliferation [55, 56]. Our findings suggested that RhoA/ROCK2 plays a proinflammatory and proproliferative role by interacting with pSTAT3 in RA-FLS. In OC differentiation, the expression levels of c-Fos, NFATc1, and other key regulators are directly related to the RhoA/ROCK2 pathway.

Studies of RA pathogenesis have shown an inseparable link between bone and the immune system [57]. Adaptive immune cell infiltration in the synovial lining is an important pathological change in RA, with half of the cells being CD4+ memory T cells [58]. The validity of CTLA4-Ig points to a relative contribution of T-cell activation even in the bone-destroying phase of RA [57]. The search for shared molecules between the skeleton and the immune system is conducive to the clinical transformation of RA therapy. As a key regulator of innate and adaptive immunity, RhoA is crucial for T-cell activation and migration [21]. The coupling of the RhoA/ROCK pathway to the JAK/STAT pathway was first discovered during differentiation of Th17 cells. Our study provides new evidence for the functional coupling between RhoA/ROCK and JAK/STAT signaling. Previous studies have shown that JAKi not only delays the bone erosion of RA, but also increases serum OPG/RANKL ratio and promotes bone mass growth in animal models of inflammatory arthritis, but the specific mechanism remains unclear [59]. The results of this study may partially explain the bone protection effect of JAKi. In summary, RhoA/ROCK2 is involved in RA-FLSs migration and proliferation, production of inflammatory mediators and metalloproteinases, expression of RANKL, and osteoclastogenesis. Precision targeted therapy is the current therapeutic trend in RA, so finding targeted pathogenic molecules that are involved in multiple aspects of the disease is of great clinical importance.

## 5. Conclusion

RhoA/ROCK2 participates in RA synovitis by coupling with the JAK2/STAT3 pathway and participates in OC bone erosion by regulating the expression of c-Fos and NFATc1 simultaneously. This study provides a theoretical basis for improving synovitis and repairing bone erosion simultaneously and lays a foundation for the development of novel RA-targeting drugs.

## Data Availability

The data used to support the findings of this study are available from the corresponding authors on reasonable request.

## Abbreviations

RA: Rheumatoid arthritis
FLS: Fibroblast-like synoviocyte
BMMC: Bone marrow mononuclear cell
RhoA: Ras homolog gene family member A
PCP: Planar cell polarity
MMPs: Matrix metalloproteinases
OPG: Osteoprotegerin
RANK: Receptor activator of nuclear factor-*κ*B
RANKL: Receptor activator of nuclear factor-*κ*B ligand
ROCK2: Rho-associated protein kinase 2
Ror2: Receptor tyrosine kinase-like orphan receptor 2
RYK: Related to tyrosine (Y) kinase
Dvl: Dishevelled
JNK: c-Jun N-terminal kinase
CIA: Collagen-induced arthritis
IHC: Immunohistochemistry
H&E: Hematoxylin and eosin
DMEM: Dulbecco's Modified Eagle Medium
*α*-MEM: *α*-Modified minimal essential medium
BMMC: Bone marrow mononuclear cell
MOI: Multiplicity of infection
CCK-8: Cell Counting Kit-8
EGFP: Enhanced green fluorescent protein
JAK2: Janus kinase 2
STAT3: Signal transducer and activator of transcription 3
NFATc1: Nuclear factor-activated T-cell 1.

## Supplementary Materials

Supplementary Materials Table S1: primers used in RT-PCR for dictation of indicated human gene. Table S2: primers used in RT-PCR for dictation of indicated mouse gene. Table S3: the manufacturers and catalog numbers of regarding antibodies used for IHC and WB.
