# Supplementary material for: RhoA Promotes Synovial Proliferation and Bone Erosion in Rheumatoid Arthritis through Wnt/PCP Pathway
Source: Mediators Inflamm. 2023 Nov 1;2023:5057009. doi: 10.1155/2023/5057009 (PMC10667059; doi:10.1155/2023/5057009)
Supplement: Supplementary Materials — Table S1: primers used in RT-PCR for dictation of indicated human gene. Table S2: primers used in RT-PCR for dictation of indicated mouse gene. Table S3: the manufacturers and catalog numbers of regarding antibodies used for IHC and WB. [file 5057009.f1.docx]

**Table S1. Primers used in RT-PCR for dictation of indicated human gene**

| Gene |  | Sequence (5’-3’) |
| --- | --- | --- |
| IL-1β | Forward primer | AGCCATGGCAGAAGTACCTG |
|  | Reverse primer | TGAAGCCCTTGCTGTAGTGG |
| TNF-α | Forward primer | CACAGTGAAGTGCTGGCAAC |
|  | Reverse primer | AGGAAGGCCTAAGGTCCACT |
| IL-17 | Forward primer | TAATGGCCCTGAGGAATGGC |
|  | Reverse primer | AGGAAGCCTGAGTCTAGGGG |
| IL-21 | Forward primer | CTGTCCTCTAGAACACACGGA |
|  | Reverse primer | TCAAAGTTGGGCAGTGAGAT |
| OPG | Forward primer | GTGTGCGAATGCAAGGAAGG |
|  | Reverse primer | CCACTCCAAATCCAGGAGGG |
| RANKL | Forward primer | CAACATATCGTTGGATCACAGCA |
|  | Reverse primer | GACAGACTCACTTTATGGGAACC |
| JNK | Forward primer | CTGAAGCAGAAGCTCCACCA |
|  | Reverse primer | CACCTAAAGGAGAGGGCTGC |
| JAK2 | Forward primer | TCTGGGGAGTATGTTGCAGAA |
|  | Reverse primer | AGACATGGTTGGGTGGATACC |
| pSTAT3 | Forward primer | CAGCAGCTTGACACACGGTA |
|  | Reverse primer | AAACACCAAAGTGGCATGTGA |
| ROCK2 | Forward primer | TCAGAGGTCTACAGATGAAGGC |
|  | Reverse primer | CCAGGGGCTATTGGCAAAGG |
| NF-κB | Forward primer | GGTGCGGCTCATGTTTACAG |
|  | Reverse primer | GATGGCGTCTGATACCACG |
| GAPDH | Forward primer | ACAACTTTGGTATCGTGGAAGG |
|  | Reverse primer | GCCATCACGCCACAGTTTC |

**Table S2. Primers used in RT-PCR for dictation of indicated mouse gene**

| Gene |  | Sequence (5’-3’) |
| --- | --- | --- |
| RhoA | Forward primer | AGCTTGTGGTAAGACATGCTTG |
|  | Reverse primer | GTGTCCCATAAAGCCAACTCTAC |
| ROCK2 | Forward primer | GGTTTACAGATGAAAGCGGAAGA |
|  | Reverse primer | GTGATGCCTTATGACGAACCAA |
| c-Fos | Forward primer | CGGGTTTCAACGCCGACTA |
|  | Reverse primer | TGGCACTAGAGACGGACAGAT |
| NFATc1 | Forward primer | GGAGAGTCCGAGAATCGAGAT |
|  | Reverse primer | TTGCAGCTAGGAAGTACGTCT |
| GAPDH | Forward primer | AGGTCGGTGTGAACGGATTTG |
|  | Reverse primer | TGTAGACCATGTAGTTGAGGTCA |

**Table S3. The manufacturers and catalogue numbers of regarding antibodies used for IHC and WB**

| Antibody | Manufacturers and Catalogue number |  |
| --- | --- | --- |
| Anti-RhoA | Abcam, ab187027, UK | IHC and WB |
| Anti-RhoA | Santa Cruz Biotechnology, sc-418, USA | IP |
| Anti-ROCK2 | Abcam, ab228000, UK | IP and WB |
| Anti-STAT3 | Abcam, ab76315, UK | IP and WB |
| Anti-MMP3 | Proteintech Group, 66338-1-Ig, China | WB |
| Anti-MMP13 | Proteintech Group, 18165-1-AP, China | WB |
| Anti-GADPH | Sigma, G9545, USA | WB |
| EGFP | Abcam, ab184601, UK | IHC |
| Anti-mouse IgG | Cell Signaling Technology, 7076, USA | WB |
| Anti-rabbit IgG | Cell Signaling Technology, 7074, USA | WB |
